# Fermented *Rhizoma* *Atractylodis Macrocephalae* alleviates high fat diet-induced obesity in association with regulation of intestinal permeability and microbiota in rats

**DOI:** 10.1038/srep08391

**Published:** 2015-02-16

**Authors:** Jing-Hua Wang, Shambhunath Bose, Hyung-Gu Kim, Kyung-Sun Han, Hojun Kim

**Affiliations:** 1 Department of Oriental Rehabilitation Medicine, Dongguk University, 814 Siksa-dong, Goyang, Gyeonggi-do, Republic of Korea; 2 Key Laboratory of Xin'an Medicine, Ministry of Education, Anhui University of Traditional Chinese Medicine, Meishan Road 103, Hefei, Anhui Province, People's Republic of China; 3 College of Pharmacy, Dongguk University-Seoul, 814 Siksa-dong, Goyang, Gyeonggi-do, Republic of Korea

## Abstract

Accumulating evidence suggests the anti-inflammatory and anti-obesity activities of *Rhizoma Atractylodis Macrocephalae* (RAM). Here, we evaluated the anti-obesity impact of unfermented (URAM) versus fermented RAM (FRAM) using both *in vitro* and *in vivo* models. Both URAM and FRAM exhibited marked anti-inflammatory, anti-adipogenic, and anti-obesity activities, and modulation of the gut microbial distribution. However, FRAM, compared to URAM, resulted in more efficient suppression of NO production and normalization of transepithelial electrical resistance in LPS-treated RAW 264.7 and HCT 116 cells, respectively. Compared to URAM, FRAM more effectively reduced the adipose tissue weight; ameliorated the serum triglyceride and aspartate transaminase levels; restored the serum HDL level and intestinal epithelial barrier function in the LPS control group. The relative abundance of *Bifidobacterium* and *Akkermansia* as well as Bacteriodetes/Firmicutes ratio in the gut of the LPS control group was significantly enhanced by both URAM and FRAM. However, FRAM, but not URAM, resulted in a significant increase in the distribution of Bacteriodetes and *Lactobacillus* in the gut of the HFD + LPS group. Our results suggest that FRAM with probiotics can exert a greater anti-obesity effect than URAM, which is probably mediated at least in part via regulation of the intestinal microbiota and gut permeability.

The etiology of obesity is known to be associated with intracellular lipid generation, excessive adipocyte accumulation, and adipose tissue storage in the whole body as a consequence of dysregulation of energy homeostasis^1^. This metabolic disease can readily enhance the risk of death through the onset of several disorders and complications, such as type 2 diabetes, hypertension, hyperlipidaemia, stroke, cardiovascular disease, musculoskeletal disorders, and colon cancer, etc.^2^. In the past decade, incidence of overweight and obesity has increased worldwide and is becoming a widespread epidemic^3^, despite development of a number of clinical anti-obesity medications for control of body weight through reduction of food intake, increase of energy expenditure, and alteration of metabolism or regulation of hormonal homeostasis^4^,^5^. Therefore, finding effective therapeutic approaches against obesity remains a challenge.

Substantial evidence indicates that the gut microbiota may play a vital role in regulation of energy balance and weight in animals and humans and may control the development and progression of obesity and other metabolic disorders^2^. A number of studies have supported that lipid metabolism is positively regulated by intestinal microbiota, principally through production of cholesterol oxidase^6^, making short chain fatty acids that inhibit liver lipase^7^ and generating conjugated bile acid hydrolase^8^. Notably, emerging evidence from animal studies strongly suggests a link between gut microbiota, increased intestinal permeability, endotoxemia, and obesity^9^. Feeding animals with a high fat diet (HFD) has been found to alter the gut microflora composition, which may be associated with an increased intestinal permeability that eventually leads to development of metabolic endotoxemia, inflammation, and metabolic disorders^10^,^11^,^12^. It has been proposed that increased intestinal permeability may lead to entry of toxins from the intestinal lumen, particularly LPS, a structural part of the gram-negative bacteria cell wall which in turn can trigger local inflammation or gain access to circulation and induce systemic inflammation through cytokine release^9^. A persistent infusion of endotoxin or a high endotoxin level induced by a high-fat diet ultimately leads to development of obesity and insulin resistance^9^. Notably, a number of studies have shown that prebiotics and probiotics can exert beneficial effects on obesity via modulation of gut microbial homeostasis. Accordingly, gut microbial environment can be considered as a potential therapeutic target for regulating development of metabolic disorders related to obesity.

*Rhizoma Atractylodis Macrocephalae* (RAM), a famed traditional herbal medicine, which has long been widely used in eastern Asia as a digestive, diuretic, and antihidrotic, represents the root of *Atractylodes Macrocephala Koidz*^13^. Previous studies also reported that RAM and its main compounds could exert multifarious pharmaceutical effects in experimental models, including anti-cancer^14^, anti-hepatotoxicity^15^, anti-coagulant^16^, anti-ulcer^17^, anti-oxidant^18^, anti-inflammation^19^, and anti-adipogenesis^20^. One study demonstrated that the water extract of RAM could exert an anti-obesity effect via Akt/PI3K pathway-modulated inhibition of lipid accumulation^20^. In addition, *Bacillus licheniformis*-fermented RAM has been reported to protect intestinal barrier function against endotoxin insult^18^, in keeping with the beneficial impact of probiotics and probiotics-mediated fermentation on the therapeutic efficacies of herbs^18^,^21^. However, so far, no detailed study evaluating the question of whether an interaction between RAM and probiotics or gut microbiota could modulate the anti-obesity impact of RAM has been reported.

As the fermentation process of herbal formulations has been found to enhance their therapeutic efficacy against a number of diseases^12^,^18^,^22^, we hypothesized that fermented herbs may be more effective than unfermented herbs against metabolic disorder. In the current study, using HFD fed rats and 3T3-L1 cells as *in vitro* and *in vivo* models, respectively, we attempted to determine whether the anti-obesity impact of RAM is associated with alteration in gut microbial environment and whether *Lactobacillus plantarum*-fermented RAM (FRAM) in association with this probiotic can exert a greater anti-obesity impact as compared with unfermented RAM (URAM). In addition, as low grade inflammation is known to be associated with the development and onset of an obese state^23^,^24^, we also evaluated the anti-inflammatory impacts of the two herbal formulations mentioned above using rats and RAW 264.7 cell line as *in vivo* and *in vitro* models for elucidation of the probable molecular mechanism(s) by which they can exert anti-obesity effects.

## Results

### URAM and FRAM did not affect cell viability under the prevailing experimental conditions

Treatment with URAM and FRAM at various concentrations (100, 200, and 400 μg/ml) for 24 h did not significantly affect the proliferation of RAW 264.7, 3T3-L1, and HCT 116 cells as compared with the corresponding normal group (Fig. S1).

### URAM and FRAM altered body and fat mass of HFD + LPS-treated rats

Treatment with both HFD and HFD plus LPS in rats resulted in significantly increased body mass and weight of abdominal, epididymal, and total fat tissues as compared with the normal group (*P* < 0.01, Table 1). Notably, exposure of rats in the HFD + LPS group to URAM or FRAM formulations resulted in significantly lowered weights of body and abdominal fat tissue, but did not cause any significant change in the weight of epididymal fat. However, exposure of rats in the HFD + LPS group to FRAM, but not URAM, resulted in a significant decrease in the weight of total fat (*P* < 0.01), and relative weights of abdominal and total fat pads (*P* < 0.05). Notably, the effect of colostrum on the body and fat pad weights of rats in the HFD + LPS group was almost similar to that of FRAM.

### URAM and FRAM ameliorated serum lipid parameters and AST activity of HFD + LPS-treated rats

HFD feeding resulted in a marked increase in the levels of serum TC, TG, and AST, and a notable reduction in the serum HDL content (*P* < 0.05 as compared to normal, Table 2). Treatment with HFD plus LPS produced a similar type of effect on the animals but with a greater extent. Exposure of HFD + LPS-treated animals to FRAM, but not URAM, resulted in significantly attenuated levels of serum TG and AST and an increase in the serum HDL content (*P* < 0.05), while the serum TC level of the HFD + LPS group was significantly decreased by treatment with both herbal formulations. On the other hand, treatment of animals in the HFD + LPS group with colostrum resulted in significantly decreased levels of serum TG and AST, but did not cause any significant change in the serum TC and HDL content.

### Evaluation of the probable molecular mechanism(s) underlying anti-adipogenic activity of URAM and FRAM *In vitro*

A decrease in adipose tissue mass of the animals in response to treatment with URAM or FRAM in our study may result from a number of events, either alone or in combination. These possibilities include decrease in adipogenesis, increase in lipolysis, decrease in lipogenesis, and apoptotic cell death. Besides, cellular uptake of glucose or changes in lipoprotein lipase (LPL) activity may also affect the adipose tissue mass. Keeping this in mind, next we attempted to understand the probable molecular mechanism(s) underlying URAM- and FRAM-mediated abatement in lipid deposition using HepG2, L6, and 3T3-L1 cell lines as *in vitro* models. Following treatment of 3T3-L1 or HepG2 cells with URAM or FRAM, we observed no significant change in Hoechst 33342 staining (Fig. S2A and S2B) and morphology of the nuclei of both cell types representing the typical hallmark of apoptosis. This finding indicates that the anti-adipogenic effect of URAM or FRAM is not mediated through adipocyte-specific apoptotic cell death. Rather, our further study on differentiated 3T3-L1 cells has revealed that the anti-adipogenic activity of URAM or FRAM is attributed, at least in part, to their ability to inhibit intracellular lipid deposition in adipocytes (Fig. S3A–3G). Although the low concentration of URAM or FRAM (100 μg/ml) only caused slightly abated intracellular lipid accumulation (approximately 2% versus control), medium and high concentrations of both of these formulations (200 μg/ml and 400 μg/ml, respectively) considerably attenuated (approximately 10%) the lipid deposition inside the cells (Fig. S3H).

Next, using 3T3-L1 and L6 cells as *in vitro* models, we assessed the effect of URAM and FRAM on the *in vitro* activity of LPL, an enzyme that plays a vital role in the catalysis of hydrolytic cleavage of triglycerides present in chylomicrons and very low density lipoproteins. For this evaluation, the activities of LPL in both the culture medium and lysate of the cells were assessed. Our results demonstrated that neither URAM nor FRAM at 100 and 200 μg/ml concentrations could produce any significant impact on LPL activity in the culture medium or homogenate of both cell types, implying that anti-adipogenic activity of these two formulations is not driven through LPL-mediated lipolysis (Fig. S4A–S4D).

Earlier studies demonstrated impaired insulin sensitivity or a state of insulin resistance in skeletal muscle of obese individuals^25^,^26^ and that an augmentation of insulin sensitivity may lead to reduction in tissue fat deposition^27^,^28^,^29^. These findings prompted us to further extend our study to assess the question of whether URAM or FRAM could improve insulin sensitivity (measured in terms of glucose uptake) in a normal or insulin resistant state. The study was conducted *in vitro* using L6 and HepG2 cells as models with the latter cells being treated with glucosamine to make them insulin resistant. Metformin, an oral antidiabetic drug known to improve insulin sensitivity, was used as a reference. Our results showed that both URAM and FRAM induced a significant increase in glucose uptake in L6 (Fig. S5A and S5B) or insulin-resistant HepG2 cells (Fig. S6A and S6B) in a concentration-dependent manner, suggesting that enhancement of insulin sensitivity may be a contributing factor through which URAM or FRAM could exert an anti-adipogenic effect.

A number of transcriptional factors including C/EBP-α and SREBP are known to be involved in the differentiation of preadipocytes into adipocytes^30^,^31^. To obtain further molecular insights into the anti-adipogenic effect of URAM and FRAM, we evaluated the impact of these two formulations on the expression of C/EBP-α and SREBP. In addition, we also examined the question of whether URAM and FRAM could exert any effect on the expression of AMPK, which was shown to accelerate glucose uptake and inhibit differentiation in 3T3-L1 preadipocytes^32^,^33^,^34^. In addition, because many 3-hydroxy-3-methyl-glutaryl-CoA reductase (HMGCR) inhibitors are known to suppress adipocyte differentiation^35^,^36^,^37^, we also investigated the question of whether URAM and FRAM could inhibit the expression of HMGCR. Our results showed that expression of C/EBP-α and HMGCR genes was significantly inhibited by URAM or FRAM at 200 μg/ml concentration (Fig. S7C and S7D). On other hand, the expression of SREBP remained unaffected in response to treatment with URAM or FRAM, although a trend was evident in the inhibition of SREBP expression by 200 μg/ml of URAM and 100 and 200 μg/ml of FRAM (Fig. S7B). A significant increase in the expression of AMPK gene was observed in the cells upon exposure to 100 and 200 μg/ml of URAM, and 200 μg/ml of FRAM (Fig. S7A).

### URAM and FRAM suppressed LPS-induced NO production in RAW 264.7 cells

Treatment with LPS resulted in a marked increase in NO production in RAW 264.7 cells (*P* < 0.05 versus normal, Fig. S8). However, exposure to FRAM or URAM resulted in significantly decreased NO production in LPS-induced cells in a concentration-dependent manner (*P* < 0.05). Notably, at 50 μg/ml concentration, this inhibitory effect was significantly more pronounced in the case of FRAM than URAM (Fig. S8).

### URAM and FRAM ameliorated HFD + LPS-induced endotoxemia

**Figure 1:**
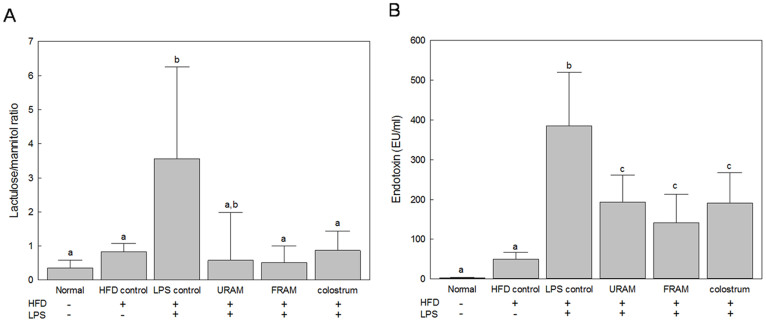
The protective effects of URAM and FRAM on the intestinal permeability and endotoxin-insult in HFD-fed rats treated with LPS. (A) Following termination of the experimental schedule, the animals were fasted for 12 h with access to water *ad libitum*. Subsequently, 1.0 ml of lactulose-mannitol solution (containing 66 mg/ml lactulose and 50 mg/ml mannitol) was administered to the animals orally. After another 20 h of fasting, urine samples were collected to determine the level of lactulose and mannitol as described in the Materials and methods section. Data are expressed as a ratio of lactulose to mannitol. (B) After termination of the experimental schedule, blood was collected from the animals and the serum endotoxin level was determined as described in the Methods section. Data are expressed as the mean ± SD (n = 8). Data with different letters are significantly different (*P* < 0.05) according to post hoc one way ANOVA analysis.

Feeding animals with HFD did not cause any significant change in serum endotoxin level (Fig. 1B). However, exposure of HFD-fed animals to LPS resulted in a dramatic elevation of the serum endotoxin content (*P* < 0.05). Notably, treatment of animals in the HFD + LPS group with URAM or FRAM resulted in significant attenuation of the serum endotoxin level (*P* < 0.05). A similar effect on the serum endotoxin content of the HFD + LPS group was shown by colostrum.

### URAM and FRAM attenuated the serum levels of CRP and inflammatory cytokines in HFD + LPS-treated rats

**Figure 2:**
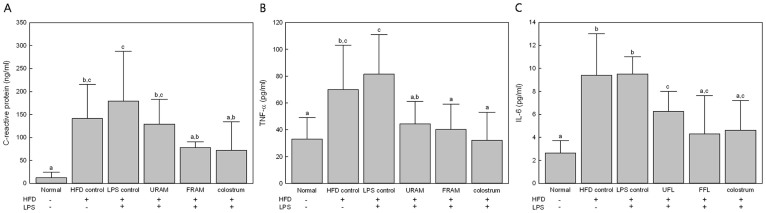
Anti-inflammatory effect of URAM and FRAM in HFD-fed rats treated with LPS. After termination of the experimental schedule, blood was collected from the animals and the serum proinflammatory cytokine levels were determined as described in the Methods section. Data are expressed as the mean ± SD (n = 8). Data with different letters are significantly different (*P* < 0.05) according to post hoc one way ANOVA analysis.

HFD-feeding or treatment with HFD plus LPS resulted in a significant increase in the serum content of CRP, TNF-α, and IL-6 (*P* < 0.05 compared with the normal group, Fig. 2). However, exposure of rats in the HFD + LPS group to URAM or FRAM resulted in markedly reduced serum levels of TNF-α and IL-6 (*P* < 0.05), whereas treatment with FRAM, but not URAM, significantly lowered the serum level of CRP in the HFD + LPS group (*P* < 0.05). On the other hand, treatment of rats in the HFD + LPS group with colostrum resulted in significantly decreased serum levels of CRP, TNF-α, and IL-6.

### URAM and FRAM protected intestinal epithelial barrier function in response to LPS-insult *in vitro* and HFD + LPS-treatments *in vivo*

Exposure to LPS resulted in markedly decreased TEER in the cell model of intestinal epithelium (*P* < 0.05 compared to normal), indicating a noticeable impairment of the epithelial barrier function (Fig. S9). Treatment of LPS-treated cells with URAM or FRAM resulted in significantly increased TEER in a concentration-dependent manner (*P* < 0.05 compared to LPS). However, augmentation of TEER in LPS-treated cells by FRAM at each concentration was significantly higher (*P* < 0.05) than that shown by FRAM at the corresponding concentration. Our *in vivo* results showed that treatment of HFD-fed animals with LPS resulted in significantly increased gut permeability in terms of an augmentation of urine L/M ratio (Fig. 1A). However, exposure of HFD + LPS-treated animals to FRAM, but not URAM, resulted in a significant decline in the L/M ratio (*P* < 0.05). A similar effect was also shown by colostrum on the animals in the HFD + LPS group.

### Distribution profile of intestinal microbiota in different experimental groups

**Figure 3:**
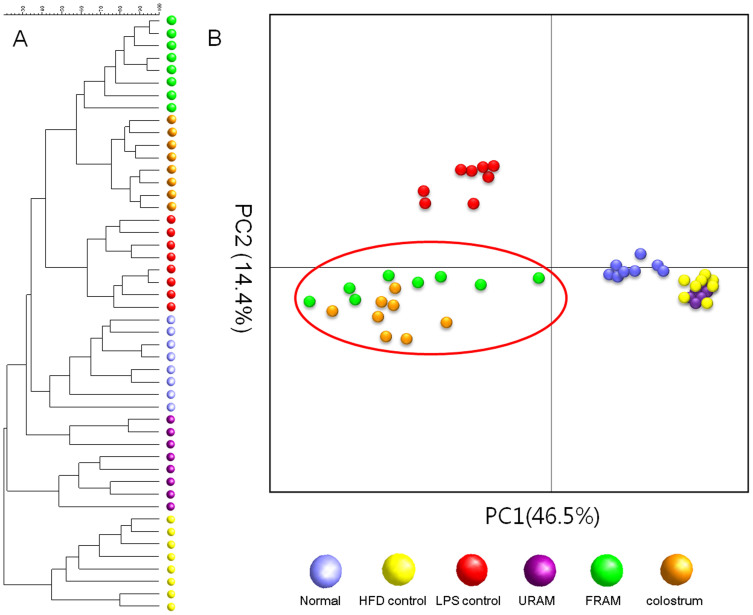
PCR-denaturing gradient gel electrophoresis fingerprinting and PCA analysis of rat stools. (A) After termination of the experimental schedule, the animals were fasted for 12 h with access to water *ad libitum*. After another 20 h of fasting, the stool samples were collected and the fecal microbial communities were analyzed by denaturing gradient gel electrophoresis as described in the Methods section. (B) PCA of the data was performed based on distance matrix (two-dimensional array) to further evaluate the similarity between bacterial communities.

**Figure 4:**
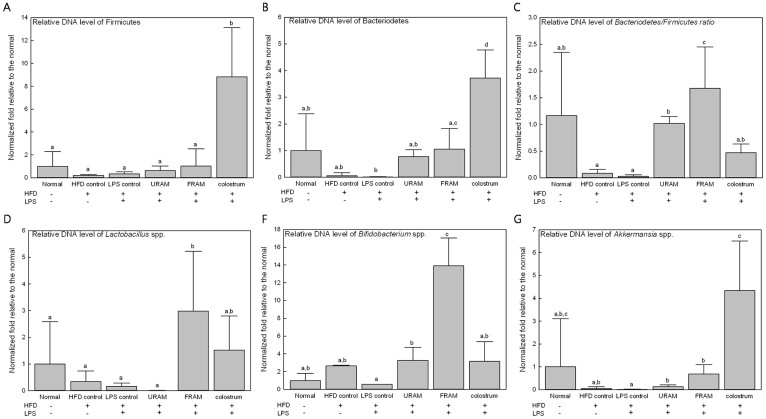
The impact of URAM and FRAM on the relative abundance of metagenomic DNA (gene-encoding 16S rRNA) of vital intestinal microbes in stools of HFD-fed rats treated with LPS. Following termination of the experimental schedule, the animals were fasted for 12 h with access to water *ad libitum*. After another 20 h of fasting, the stool samples were collected and the abundance of the 16S rRNA gene of the bacterial strains was determined as described in the Methods section. The results are expressed as normalized fold values relative to the normal group. Data are expressed as the mean ± SD (n = 8). Data with different letters are significantly different (*P* < 0.05) according to post hoc one way ANOVA analysis.

Treatment of animals in the HFD + LPS group with URAM or FRAM resulted in an observable alteration in the distribution of gut microbial community, as indicated by the distinct clustering patterns in the denaturing gradient gel electrophoresis profile and principal component analysis (PCA) analysis (Fig. 3A and 3B). More specifically, a close association of denaturing gradient gel electrophoresis pattern and PCA analysis was observed between the HFD control and HFD + LPS + URAM groups as well as between the HFD + LPS + FRAM and colostrum groups (marked with a red circle in Fig. 3B). To further analyze the distribution profile of the gut flora in detail and to investigate the relationship between the herbal formulations and intestinal microbial niche, the abundance of several vital strains of gut flora, including *Lactobacillus* spp., *Bifdobacterium* spp., *Akkermansia* spp., Bacteriodetes, and Firmicutes in the stool was evaluated using quantitative real time-PCR. Accordingly, HFD-feeding or treatment with HFD plus LPS resulted in a statistically insignificant, but considerable reduction in the relative abundance of *Lactobacillus* spp., *Akkermansia* spp., Bacteriodetes, and Firmicutes, as well as the Bacteriodetes/Firmicutes ratio (Fig. 4). On the other hand, the relative abundance of *Bifidobacterium* spp. was augmented by HFD feeding but decreased by HFD feeding plus LPS treatment, both in an insignificant manner. Of particular interest, the relative abundance of *Bifidobacterium* spp. and *Akkermansia* spp. as well as the Bacteriodetes/Firmicutes ratio in the HFD + LPS group was significantly enhanced upon co-treatment with URAM or FRAM (*P* < 0.05 or 0.01; Fig. 4), whereas the relative abundance of Bacteriodetes and *Lactobacillus* spp. in the HFD + LPS group was significantly augmented by FRAM (*P* < 0.05), but not by URAM (Fig. 4).

## Discussion

A considerable number of traditional herbal medicines have long been used in eastern Asia for treatment of various metabolic disorders, including obesity and non-insulin dependent diabetes mellitus (NIDDM)^38^. However, lack of adequate scientific knowledge and evidence to explain the mechanism of action restricts the popularization and application of these medications. Using various experimental models, previous studies have demonstrated the medicinal effects of *Rhizoma Atractylodis Macrocephalae* (RAM) against a number of diseases, including obesity^14^,^15^,^16^,^17^,^18^,^19^,^20^.

The beneficial effects of probiotics and their fermented food products on health are well documented^39^. The fermentation process has been shown to improve biological properties of plants, vegetables, and herbs^18^,^22^,^40^,^41^,^42^. The current study was conducted in order to understand the detailed molecular mechanism(s) underlying the anti-obesity impact of RAM using both *in vivo* and *in vitro* experimental models. In addition, another goal of our study was to determine whether the anti-obesity property of RAM is associated with alteration in gut microbial environment and whether *Lactobacillus plantarum*-fermented RAM in association with this probiotic can exert a greater anti-obesity effect in comparison with unfermented RAM.

A number of previous studies have suggested that host-gut microbial metabolic interactions can play a significant role in predisposal to obesity^43^,^44^,^45^, which is driven by an augmentation in the capacity of energy harvest from the diet and induction of low-grade chronic systemic inflammation^46^,^47^,^48^,^49^. Our results showed significantly elevated levels of serum endotoxin, TNF-α, and IL-6 in animals fed a HFD-diet for eight weeks compared to those fed a normal diet for the same time period. Earlier studies demonstrated that HFD can induce low-grade inflammation through an increase in the endotoxin level resulting from modulation in intestinal microbiota and augmentation of intestinal permeability^12^,^49^. The intestinal derived endotoxin (LPS, the main component of gram-negative bacteria outer membrane) stimulates inflammatory cells, such as lymphocytes and macrophages, etc., subsequently resulting in secretion of inflammatory mediators, such as NO, TNF-α, IL-6, and CRP^50^,^51^,^52^.

In the current study, both URAM and FRAM exhibited *in vitro* anti-inflammatory activity as demonstrated by their ability to significantly attenuate LPS-induced production of NO in RAW 267.4 cells without affecting cell viability. The anti-inflammatory activities of URAM and FRAM were further confirmed in our *in vivo* study, where both of these formulations caused significant depletion of the level of serum endotoxin, TNF-α, and IL-6 in animals of the HFD + LPS group. However, in the above mentioned studies, FRAM exhibited a significantly higher inhibition of NO production compared to RAM at 50 μM concentration, and exposure of animals in the LPS + HFD group to FRAM but not URAM, resulted in a significantly reduced level of CRP, a critical protein related to bacterial infection and inflammation^53^. Based on these findings, it is conceivable that fermented RAM may be more effective than unfermented RAM in attenuation of systemic inflammation.

Next we attempted to determine whether URAM and FRAM could exert an anti-obesity impact utilizing both *in vitro* and *in vivo* models. In our *in vivo* study, feeding animals with HFD for eight weeks induced severe obesity, as indicated by a significant increase in body weight, as well as abdominal (and epididymal fat weight, accompanied by elevation of the levels of serum TC, TG, and AST and reduction of the content of serum HDL. Co-treatment with a single dose of LPS (0.75 mg/kg) in HFD-fed animals resulted in further enhancement of the obesity impact, as reflected by higher levels of serum AST and TG and a lower content of serum HDL in the HFD + LPS group compared with the HFD control group. These findings are indicative of the induction of metabolic dysfunction in the animals in response to HFD feeding and LPS treatment, consistent with previous reports^40^,^49^,^54^. Nonetheless, exposure of HFD + LPS-treated animals to URAM or FRAM resulted in significantly decreased body mass and abdominal fat weight and significant attenuation in the level of total cholesterol. Of particular importance, treatment of rats in the HFD + LPS group with FRAM, but not URAM, resulted in a significant decrease in the relative weight of abdominal fat, total fat mass as well as relative weight of total fat, a significant reduction in the level of serum TG and activity of AST, and a significant increase in the level of serum HDL. Considering all of these results, it is conceivable that fermented formulation of RAM is more effective than unfermented RAM in combating obesity through promotion of lipid metabolism.

A reduction in adipose tissue mass as observed above may be the consequence of a number of events, either alone or in combination. These possibilities include decline in adipogenesis, augmentation of lipolysis, reduction in lipogenesis, and apoptotic cell death. Besides, cellular uptake of glucose or alteration in lipoprotein lipase activity may also influence the adipose tissue mass. In our *in vitro* experiment, treatment with both URAM and FRAM resulted in a marked suppression of lipid accumulation in differentiated 3T3-L1 cells, which are widely used as a model of adipogenesis^55^ due to their potential capacity for differentiation from fibroblasts to adipocytes^56^. Previous studies have reported that RAM and its active components, including atractylenolide I, atractylenolide II, and atractylon inhibit adipogenesis via the PI3K/Akt signaling pathway^20^,^57^. Notably, in our study, no marked difference in the degree of inhibition of lipid accumulation was observed between URAM and FRAM-treated cells, implying that fermentation-mediated constitutional changes did not improve the anti-adipogenic activity of RAM.

Our further *in vitro* studies on 3T3-L1 and L6 cells have revealed that the anti-adipogenic effect of URAM or FRAM was neither driven through apoptotic cell death nor contributed by LPL-mediated lipolysis. Notably, a number of studies have reported decreased insulin sensitivity or a state of insulin resistance in skeletal muscle of obese individuals^25^,^26^. Association of insulin resistance in obesity and type 2 diabetes with decreased insulin-stimulated glucose transport and metabolism in adipocytes and skeletal muscle as well as impaired inhibition of hepatic glucose output has been demonstrated^58^,^59^. Based on earlier studies, it is conceivable that an improvement in insulin sensitivity (or increase in glucose uptake) may result in a reduction of tissue fat deposition^27^,^28^,^29^. Notably, in our *in vitro* study, a significant increase in glucose uptake by L6 or insulin-resistant HepG2 cells was observed in response to treatment with URAM or FRAM in a concentration-dependent manner, indicating that these two formulations can augment insulin sensitivity. An earlier study on 3T3-L1 cells showed that atractylodes could promote glucose transport by increasing the levels of glucose transporter 4 (GLUT-4), phosphatidylinositol 3-kinase (PI3K), and insulin receptor substrates-1 (IRS-1)^60^.

Substantial evidence indicates that multiple transcriptional factors including C/EBP-α (CCAAT-enhancer-binding protein alpha) and SREBP (sterol regulatory element binding protein) play a vital role in the differentiation of preadipocytes into adipocytes^30^,^31^. Our results showed that in 3T3-L1 cells, expression of C/EBP-α, but not SREBP was significantly downregulated by URAM or FRAM. In addition, our further studies showed that both of these formulations induced significantly upregulated expression of AMPK (5′ adenosine monophosphate-activated protein kinase), the activation of which is known to promote glucose uptake and suppress differentiation in 3T3-L1 preadipocytes^32^,^33^,^34^. These findings are in keeping with an earlier study demonstrating that *Rhizoma Atractyloidis* acupuncture in HFD-induced obese ICR mice inhibited expression of a number of proteins including C/EBP-α and enhanced the activation of AMPK associated with the inhibition in development of weight gain, hyperglycemia, hyperinsulinemia, and hyperlipidemia as well as the enlargement of fat cell size^61^. In addition, we also found that the expression of HMGCR whose inhibition is known to suppress adipocyte differentiation^35^,^36^,^37^ was significantly attenuated by URAM or FRAM. The inhibitory effect of herbal extract on hepatic expression of HMGCR was shown in a previous study on HFD-fed mice^62^.

Previous study has shown that feeding animals with HFD alters the gut microbial population^10^ and that modulation of gut microbiota is associated with an increased intestinal permeability^11^. Augmentation of gut permeability promotes LPS absorption in serum, ultimately leading to endotoxemia, a condition that triggers inflammation and various metabolic disorders^11^. Endotoxemic insult is also known to enhance gut permeability through destabilization of intestinal barrier integrity^11^,^63^, in agreement with our TEER studies, where intestinal epithelial cells showed impaired barrier function in response to LPS-treatment. Notably, exposure of LPS-treated cells to URAM or FRAM resulted in significantly augmented TEER. However, increase of TEER in LPS-treated cells by FRAM at each concentration was significantly higher than that shown by FRAM at the corresponding concentrations. The above mentioned findings are also consistent with those of our *in vivo* study, where the urine L/M ratio, a valuable indicator of gut permeability^64^, was significantly elevated in parallel with a significant increase in serum endotoxin level in the animals in response to HFD feeding and LPS insult together. Of particular importance, co-treatment of animals in the HFD + LPS group with URAM or URAM resulted in significant attenuation of the serum endotoxin level. However, exposure of HFD + LPS-treated animals to FRAM, but not URAM, resulted in a significant decrease in the L/M ratio. Thus, based on our findings it is conceivable that both URAM and FRAM can combat HFD or HFD + LPS-induced inflammation probably via reduction of the serum endotoxin content and attenuation of the production or release of pro-inflammatory cytokines. However, FRAM appeared to be more effective than URAM *in vivo* in counteracting with serum endotoxin and exerting a beneficial impact on the membrane permeability of intestinal epithelial cells both *in vitro* and *in vivo*.

In mammals, it has been estimated that more than 10 trillion microbes colonize in the gastrointestinal tract throughout their life span^65^,^66^. Commensal microorganisms play an important role in promoting digestion of food and nutrient absorption, regulation of intestinal permeability, and prevention of tumor formation, etc. via interaction with the host^67^,^68^,^69^. Relationship of HFD-induced obesity with changes in gut microbiota and gut inflammation has been reported in a diet-induced obese animal model^54^. In keeping with this, previous studies have found that an imbalance in the intestinal microbial population contributes to development of obesity, which is mediated through promotion of energy harvest from diet, induction of systemic inflammation, and acceleration of fat deposition^70^,^71^.

Notably, in the human digestive tract, almost all intestinal microbiota (approximately 99%) are derived from five types of phyla, Firmicutes, Bacteroidetes, Actinobacteria, Fusobacteria, and Proteobacteria^45^. Of particular interest, reduction in both the relative proportion of Bacteroidetes and the ratio of Bacteroidetes to Firmicutes is perceptibly associated with an increase in body weight^45^,^69^,^72^. In addition, several lines of evidence indicate that numerous species of gut bacteria can interfere with development and onset of obesity. For example, a beneficial anti-obesity effect was reported to be exerted *in vivo* by various species of *Lactobacillus*, including *Lactobacillus plantarum*^73^,^74^, *Lactobacillus gasseri*^75^, *Lactobacillus Casei Shirota*^76^, etc., whereas *Bifidobacterium* strains were found to affect HFD-induced fat distribution^77^ and *Akkermansia muciniphila* was shown to reverse HFD-induced obesity, metabolic endotoxemia, and improve glucose homeostasis^48^,^78^.

In the current study, in gross, FRAM was evidently found to exert a greater beneficial impact compared to URAM. To determine whether this extra beneficial effect of FRAM is influenced by the gut microbial population, and if so, whether it is related to a specific bacterial community, we performed PCR-denaturing gradient gel electrophoresis analyses of the stool samples of animals. Accordingly, cluster analyses of PCR-denaturing gradient gel electrophoresis fingerprints indicated that co-treatment with FRAM or colostrum led to modification of the pattern of bacterial community in HFD-fed plus LPS-treated animals. In addition, PCA analyses of denaturing gradient gel electrophoresis fingerprints revealed a distinctly separate cluster for the URAM + HFD + LPS group, which showed a close association with that of the HFD group. In addition, the pattern of clusters also indicated a close association of the FRAM + HFD + LPS group with the colostrum + HFD + LPS group. Based on these findings, it is conceivable that FRAM, but not URAM, can impose a prebiotic effect, which is analogous with colostrum. In order to obtain a further detailed understanding of the role of interaction between RAM and gut microbes in alleviating obesity, several critical phyla and genera of intestinal flora in the stool samples were quantified using real-time PCR. Our results demonstrated that HFD-feeding or treatment with HFD plus LPS resulted in a statistically insignificant, but a marked reduction in the relative abundance of *Lactobacillus* spp., *Akkermansia* spp., Bacteriodetes, and Firmicutes, as well as the Bacteriodetes/Firmicutes ratio. However, the relative abundance of *Bifidobacterium* spp. and *Akkermansia* spp. as well as the Bacteriodetes/Firmicutes ratio in the HFD + LPS group was significantly increased by co-treatment with both URAM and FRAM, while the relative abundance of Bacteriodetes and *Lactobacillus* spp. in the HFD + LPS group was significantly elevated by FRAM, but not by URAM. These results indicate a marked beneficial impact of the RAM formulations, particularly FRAM, on the distribution profile of intestinal bacteria that are known to influence obesity.

In conclusion, treatment with fermented RAM resulted in more prominent improvement in HFD-induced obesity than RAM. Based on our *in vitro* and *in vivo* studies, it is conceivable that anti-adipogenic and anti-obesity impact of FRAM may be mediated, at least in part, via suppression of adipogenesis by a number of mechanisms, improving glucose uptake, influencing the distribution of the intestinal microflora accompanied by improvement in gut permeability and prevention of endotoxemia and associated inflammation. Future in-depth studies are needed in order to identify the compound(s) and mediator(s) present in the fermented RAM that may account for providing the beneficial properties of FRAM.

## Methods

### Bacterial culture

The stock inoculum of *Lactobacillus plantarum* was a generous gift from Cell Biotech (Gimpo, South Korea). Before performing herbal fermentation, the bacterial stock was repeatedly sub-cultured at 37°C for 48 h in *Lactobacilli* MRS broth (Difco-BD, Sparks, MD, USA) under anaerobic and sterile conditions.

### Preparation and fermentation of aqueous extract of RAM

RAM (Korean Pharmacopoeia standard grade) was purchased from Dongguk International Hospital (Goyang, South Korea), and was identified by Prof. Je-Hyun Lee (College of Oriental Medicine, Dongguk University, South Korea). The extraction and fermentation of the herb was performed following our lab-optimized procedure as described previously^7^. Briefly, dried powder (50 g) of RAM was dissolved in 500 ml of distilled water and, following sonication for 30 min, the mixture was incubated for 3 h at 70°C. The resultant product was then autoclaved for 15 min at 121°C for sterilization and decoction. Subsequently, the herbal extract was inoculated with 2 × 10^7^ colony forming units (CFU)/ml of *Lactobacillus plantarum*. The mixture was then incubated for 24 h at 37°C to produce the FRAM where the starter bacterial population was kept alive further in order to achieve the probiotic effects. At the end of fermentation, we confirmed the achievement of optimal fermentation by measuring CFU of *Lactobacillus plantarum* and pH of the FRAM preparation. For this validation, the bacterial count should be over 7 × 10^9^ CFU/ml and pH of the formulation should reach a range of 4.0–4.5. The rationale behind the selection of *Lactobacillus plantarum* for the fermentation process is based on our pre-experimental findings, which indicated that this bacterial strain is the most suitable for RAM fermentation compared to other bacterial spp., such as *Bacillus licheniformis*, *Lactobacillus brevis*, *Lactobacillus acidophilus* etc. (data not shown). URAM was prepared in a similar way, except that 2% (v/v) of the sterile Lactobacilli MRS broth was added instead of the bacterial inoculum. Finally, extracts were subjected to filtration using cheese cloth and freeze dried at −80°C for 72 h. The yielded products were stored at −70°C for future use.

### Animals and experimental design

Male Sprague-Dawley (SD) rats (180–220 g body weight) were obtained from Orient Bio (Seongnam, South Korea). After seven days of acclimation in controlled conditions of temperature (22 ± 2°C), relative humidity (40%–60%), and a 12 h light-dark cycle (lights on at 7:00 Am) with access to normal diet (AIN-93G, FEEDLAB, Kyonggi-do, South Korea) and water *ad libitum*, animals were randomly divided into six groups of eight rats each. The groups were as follows: normal, HFD control (high fat diet only), LPS control (HFD + 0.75 mg/kg of LPS), URAM (250 mg/kg of URAM + HFD + 0.75 mg/kg of LPS), FRAM (250 mg/kg of FRAM + HFD + 0.75 mg/kg of LPS), and colostrum control (10% colostrum + HFD + 0.75 mg/kg of LPS). Due to the well-known intrinsic property of natural prebiotics, colostrum, also called beestings, was applied as a reference control. During the entire experimental period of eight weeks, the normal group was given normal chow while a high fat diet (FEEDLAB, Kyonggi-do, South Korea) was fed to animals in all other groups. Both detailed formulation and ingredients of normal diet and high fat diet are shown in Table S1. From the sixth week onward, animals in the respective groups were treated with URAM (250 mg/kg), FRAM (250 mg/kg), and colostrum (1 ml/rat, 10% of a commercial product, Colostrum Technologies GmbH, Germany) by oral gavage once per day for a period of two weeks, whereas, instead of herbal drugs, distilled water was administered to animals in the normal and HFD control groups. At the end of the eighth week, only a single dose of LPS (0.75 mg/kg) was administered i.p. to animals in all groups, except for those in the normal and HFD control groups. At the 12th h of LPS injection, animals in all groups were transferred to individual metabolic cages and fasted for 12 h with access to water *ad libitum*. Subsequently, 1 ml of solution of lactulose/mannitol mixture (containing 66 mg/ml lactulose and 50 mg/ml mannitol in water) was administered to the animals by oral gavage. After fasting for another 20 h, stool and urine samples were collected and stored at −70°C for future analyses; all animals were then weighed and sacrificed. Blood was collected from the abdominal aorta under anesthesia and the abdominal and epididymal fat pads were excised and weighed. All experimental procedures, including the care and handling of animals, were performed following the international guidelines. (Guide for the Care and Use of Laboratory Animals, Institute of Laboratory Animal Resources, Commission on Life Sciences, National Research Council, National Academy Press, Washington, DC, USA, 1996.)^79^ The rationale, design, and protocols of this study were approved by the Institutional Animal Ethical Committee, Dongguk University, South Korea. (No. 2011-1268). Clinically, raw RAM is generally used at a dose of 15 g/60 kg/day. Following the US FDA guidelines, we converted the human dose of RAM to an animal equivalent dose based on body surface area and the yield of herbal extract.

### Analyses of serum biochemical parameters

Serum was separated from blood using Vacutainer tubes (BD, Plymouth, UK) after clotting at room temperature for 1 h. The serum levels of total cholesterol (TC), high density lipoprotein (HDL), triglyceride (TG), and aspartate transaminase (AST) were determined using respective commercial kits from Asan Pharmaceutical (Seoul, South Korea). The level of serum endotoxin was determined using a Limulus Amebocyte Lysate (LAL) kit (ENDOSAFE, SC, USA) according to our previous report^12^ and following the kit manufacturer's protocol.

### Determination of lactulose and mannitol in urine

For evaluation of the intestinal passive permeability of the animals administered lactulose (L) and mannitol (M), the urinary levels of these two sugars were measured using K-Lactul and K-Manol kits (Megazyme, Wicklow, Ireland) according to our previous report^12^ and following the kit manufacturer's instructions. The results were expressed as a ratio of lactulose to mannitol (L/M).

### Determination of serum levels of proinflammatory cytokines

The levels of C-reactive protein (CRP), tumor necrosis factor-alpha (TNF-α), and interlukin-6 (IL-6) in serum were measured using respective rat-specific commercial ELISA kits (USCN, TX, USA for CRP; BioLegend, San Diego, CA, USA for TNF-α and IL-6) according to the kit manufacturer's instructions.

### Fecal microbiological analysis using denaturing gradient gel electrophoresis and quantitative real-time PCR (qRT-PCR)

Genomic DNA from the rat stool was isolated using an AccuPrep stool DNA extraction kit (Bioneer, Daejeon, South Korea), which was subsequently analyzed in order to determine the pattern and relative abundance of the intestinal microbial community. The stool metagenomic DNA and universal bacterial primer based on 16s rRNA genes (27F, 5′-AGA GTT TGA TCC TGG CTC AG-3′ and 1525R, 5′-AAG GAG GTG ATC CAG CC-3′, Bioneer, Daejeon, South Korea) were used in performance of the routine PCR procedure. After electrophoresis of the first PCR products in 1% agarose gel and subsequent staining with ethidium bromide (EB, Bio-Rad, CA, USA), only up to 1.5 kb bands of the gels were cut and DNA was extracted using an Accuprep gel purification kit (Bioneer). The second round of PCR was performed using a primer with a GC-clamp (314f-GC, 5′-CGC CCG CCG CGC GCG GCG GGC GGG GCG GGG GCA CGG GGG GCC TAC GGG AGG CAG CAG-3′ and 518r, 5′-ATT ACC GCG GCT GCT GG-3′), which amplified the V3 region of 16S rDNA. Subsequently, denaturing gradient gel electrophoresis analysis of the samples was performed using a D-Code universal mutation detection system (Bio-Rad, CA, USA). For this, the second PCR products (20 μl) were loaded on 40% Acrylamide/bis (37.5:1) gels with a denaturing gradient maintained at 35–57.5% (where 100% is 7 M urea and 40% (v/v) formamide) in 1 × TAE buffer (ViroMed, Seoul, South Korea). Electrophoresis was performed at 80 V at a temperature of 60°C for 15 h. After staining with ethidium bromide at room temperature for 10 min, the gels were photographed under UV illumination (LAS-3000; Fuji photo film, Tokyo, Japan). The pattern of denaturing gradient gel electrophoresis in gels was analyzed using Bionumerics 3.0 software (Applied Maths, Sint-Martens-Latem, Belgium) by selection of an unweighted pair group method and an arithmetic means (UPGMA) clustering procedure based on genetic similarity expressed by the Dice coefficient. In addition, PCA of the denaturing gradient gel electrophoresis profiles in the samples was also performed.

qRT-PCR of the stool DNA samples was performed using a LightCycler instrument (Roche Applied Science, Indianapolis, ID, USA) and a lightCycler FastStart DNA Master SYBR Green kit (Roche Applied Science). The primers (COSMO GENETCH, Seoul, Korea) used in targeting the 16S rRNA gene of the bacteria are shown in Table S2^48^,^80^,^81^. The optimization of annealing temperatures of the primers and standardization of the conditions for real-time PCR amplification reactions were carried out as previously described^22^. LightCycler software (Roche Applied Science) was used for analysis and interpretation of the data. Relative abundance of bacterial phylum or genus was represented by 2^−ΔC^, where C_t_ denotes the crossing threshold value as derived by the software. The final results are expressed as normalized fold values relative to the normal group.

### Statistical analyses

All experimental results are expressed as the mean ± SD (standard deviation). The statistical package for social science (SPSS, 17.0 version, Chicago, IL, USA) software was applied for statistical analyses of the data. Statistical significance of difference was determined using one way ANOVA followed by LSD (least significant difference) post-hoc test. The difference was considered statistically significant when *P* < 0.05.

## Author Contributions

H.J.K. designed the study; H.G.K. performed the animal experiments; J.H.W. performed the *in vitro* experiments; H.G.K. and K.S.H. analyzed the data; J.H.W. and S.B. wrote the manuscript.

## Supplementary Material

Supplementary Information Supplementary Information

## Figures and Tables

**Table 1:** Comparison of body and fat masses

High Fat Diet	−	+	+	+	+	+
LPS (0.75 mg/kg)	−	−	+	+	+	+
Groups	Normal	HFD control	LPS control	URAM	FRAM	Colostrum
Body mass (g)	355 ± 27	465 ± 33##	457 ± 18$$	409 ± 35*^,^&	384 ± 53**^,^&	398 ± 59*^,^&
Abdominal fat (g)	6.1 ± 1.2	21.1 ± 9.0##	24.1 ± 4.9$$	16.3 ± 6.6&	15.7 ± 4.8&&	13.0 ± 4.2&&
Rela. of Abf (%)	1.9 ± 0.4	5.0 ± 1.7##	5.3 ± 1.2$$	3.9 ± 2.1	3.8 ± 1.1&	3.1 ± 1.0*^,^&&
Epididymal fat (g)	5.4 ± 0.9	11.4 ± 3.0##	12.7 ± 3.4$$	11.4 ± 3.4	9.9 ± 2.4	9.5 ± 3.0
Rela. of Epf (%)	1.5 ± 0.2	2.5 ± 0.6##	2.8 ± 0.8$$	2.8 ± 1.1	2.7 ± 0.7	2.3 ± 0.6
Total fat (g)	11.5 ± 1.5	32.4 ± 11.6##	36.7 ± 7.2$$	27.8 ± 9.8	22.8 ± 4.0&&	22.6 ± 7.0&&
Rela. of fat (%)	3.3 ± 0.5	7.0 ± 2.6##	8.1 ± 1.8$$	6.8 ± 3.0	6.0 ± 1.4&	5.6 ± 1.1&

Abbreviations: Rela. of Abf, relative weight of abdominal fat; Rela. of Epf, relative weight of epididymal fat; Rela. of fat, relative weight of total fat; URAM, unfermented *Rhizoma Atractylodis Macrocephalae*; FRAM, fermented *Rhizoma Atractylodis Macrocephalae*. Body weights were measured after 18 h of LPS injection before sacrifice, and relative fat weight was calculated as fat weight/body weight. Data were expressed as Mean ± SD and statistically evaluated using one way ANOVA followed by LSD pos-hoc test.

^##^*P* < 0.01, compared to the normal group;

*P < 0.05,

**P < 0.01 compared to the HFD control group;

^$$^*P* < 0.01, compared to the normal group;

^&^*P* < 0.05,

^&&^*P* < 0.01 compared to the LPS control group (n = 8).

**Table 2:** Comparison of serum biochemical parameters

High Fat Diet	−	+	+	+	+	+
LPS (0.75 mg/kg)	−	−	+	+	+	+
Groups	Normal	HFD control	LPS control	URAM	FRAM	Colostrum
TC (mg/dl)	46.1 ± 5.4	67.1 ± 14.7#	82.2 ± 18.2$$	64.7 ± 10.8&	60.4 ± 10.5&	65.6 ± 11.1
HDL (mg/dl)	38.2 ± 4.9	29.6 ± 5.9#	21.5 ± 4.4*^,^$$	26.7 ± 5.3*	28.7 ± 6.4&	26.4 ± 5.2
TG (mg/dl)	28.1 ± 7.8	42.1 ± 12.7#	78.2 ± 33.3*^,^$	55.8 ± 10.8	42.8 ± 6.3&	42.7 ± 17.8&
AST (IU/l)	27.2 ± 9.3	50.4 ± 25.6#	124.4 ± 63.4*^,^$$	84.6 ± 30.9*	58.6 ± 22.3&	13.2 ± 1.6**^,^&&

Abbreviations: TC, total cholesterol; HDL, high density lipoprotein; TG, triglyceride; AST, aspartate transaminase; URAM, unfermented *Rhizoma Atractylodis Macrocephalae*; FRAM, fermented *Rhizoma Atractylodis Macrocephalae*. Data were expressed as Mean ± SD and statistically evaluated using one way ANOVA followed by LSD pos-hoc test.

^#^*P* < 0.05, compared to the normal group;

*P < 0.05,

**P < 0.01 compared to the HFD control group;

^$^*P* < 0.05,

^$$^*P* < 0.01, compared to the normal group;

^&^*P* < 0.05,

^&&^*P* < 0.01 compared to the LPS control group (n = 8).
