# Supplementary material for: Fermented Rhizoma Atractylodis Macrocephalae alleviates high fat diet-induced obesity in association with regulation of intestinal permeability and microbiota in rats
Source: Sci Rep. 2015 Feb 16;5:8391. doi: 10.1038/srep08391 (PMC4329570; doi:10.1038/srep08391)
Supplement: Supplementary Information [file srep08391-s1.pdf]

Supplementary Information

Fermented *Rhizoma Atractylodis Macrocephalae* alleviates high fat diet-induced obesity in association with regulation of intestinal permeability and microbial population in rats

Running title: Anti-obesity effect of fermented *Rhizoma Atractylodis Macrocephalae*

Jing-Hua Wang[1, 2#], Shambhunath Bose [3#], Hyung-Gu Kim[1], Kyung-Sun Han[1], Hojun Kim[1*]

[1]Department of Oriental Rehabilitation Medicine, Dongguk University, 814 Siksa-dong, Goyang, Gyeonggi-do, Republic of Korea

[2]Key Laboratory of Xin'an Medicine, Ministry of Education, Anhui University of Traditional Chinese Medicine, Meishan Road 103, Hefei, Anhui Province, People's Republic of China

[3]College of Pharmacy, Dongguk University-Seoul, 814 Siksa-dong, Goyang, Gyeonggi-do, Republic of Korea

# These authors contributed equally.

* Corresponding author: Hojun Kim

Department of Oriental Rehabilitation Medicine, Dongguk University-Seoul, Graduate School of Oriental Medicine 814 Siksa-dong, Goyang city, Gyeonggi-do, Republic of Korea Tel: +82 31 961 9101/+82 31 961 9111; Fax: +82 31 961 9009; E-mail: kimklar@gmail.com

Supplementary methods

Cell culture and cell viability assay. RAW 264.7 (murine leukemic monocyte-macrophages) and 3T3-L1 (murine preadipocytes), L6 myocytes (rat myoblasts), and HepG2 (human hepatocarcinoma cells) (Korea Cell Line Bank, Seoul, Korea) were maintained and cultured in Dulbecco's Modified Eagle Medium (DMEM, GIBCO, CA, USA) supplemented with 10% fetal bovine serum (FBS, GIBCO, CA, USA), 2 mM L-glutamine (GIBCO, CA, USA), 100 U/ml penicillin (GIBCO, CA, USA), and 100 µg/ml streptomycin (GIBCO, CA, USA). HCT 116 (colorectal carcinoma epithelial cells) was cultured in McCoy's 5A medium (Invitrogen, CA, USA) containing 10% FBS, 2 mM L-glutamine, 100 U/ml penicillin, and 100 µg/ml streptomycin. Culturing of all cell lines was performed at 37˚C in a humidified atmosphere in an environment of 5 % CO_2.

The cell viability of RAW 264.7, 3T3-L1, and HCT-116 cells was measured colorimetrically using an EZ-cytox enhanced cell viability assay kit (DOGEN, Seoul, Korea). Briefly, after achieving around 80% confluency, the cells were treated for 24 h with URAM or FRAM at 100, 200, and 400 µg/ml concentrations or water (for the normal group). EZ-Cytox was added to the cells 2 h prior to the end of the treatment schedule. Following completion of the reaction, the culture media were transferred to a new 96-well microplate. The absorbance of the wells was read at 450 nm (650 nm as a reference wavelength) (Spectramax Plus, Molecular Devices, CA, USA). The viability of the control cells, in terms of their absorbance, was set to 100%.

Detection of apoptosis through propidium iodide (PI) and Hoechest 33342 double staining of cells *in vitro.* 3T3-L1 and L6 cells were seeded at a density of 2×10^5 cells per well into 6-well plates. Following an overnight culture, the cells were treated with URAM or FRAM at 100 and 200 µg/mL concentrations for 24 h. Following this, the cells were washed thoroughly with PBS, pH 7.4, and then stained with 5 µg/ml of PI (Sigma-Aldrich, MO, USA) for 10 min at 37°C. The cells were then co-stained with 15 µg/ml Hoechst 33342 for 10 min at 4°C. Finally, the cells were observed under fluorescence microscopy (Olympus BX-61) using a mercury vapor lamp for epiillumination and appropriate excitation and emission filter sets for monitoring PI and Hoechst 33342 staining. The images were acquired using an Olympus DP70 digital camera.

Preadipocyte differentiation and oil red O staining *in vitro*. 3T3-L1 cells were seeded in 96-well plates at a density of 3 × 10^3 cells/well. After proliferation for 48 h, the cells were treated with 1 μM dexamethasone (Sigma-Aldrich, MO, USA), 0.5 mM 3-isobutyl-1-methylxanthine (IBMX, Sigma-Aldrich), and 10 μg/ml insulin (Eli Lilly and company, IN, USA) in DMEM containing 10% FBS for 72 h. Subsequently, the media was changed every 48 h using DMEM supplemented with 10% FBS and 10 μg/ml insulin. When the intracellular accumulation of lipid droplets was prominently visible, the cells were treated with URAM or FRAM (0, 100, 200, and 400 μg/ml) for 24 h.

After fixation in 10% formalin for 1 h, the differentiated 3T3-L1 cells were washed with 60% isopropanol and then stained with Oil red O (Sigma-Aldrich, MO, USA) for 10 min. The cells were observed under an Olympus BX61 inverted microscope (Olympus, Tokyo, Japan) and images were captured using a DP70 digital camera (Olympus, Tokyo, Japan). The cells were then treated with 100% isopropanol for 10 min and the resultant supernatant was collected. The absorbance of the supernatant was read at 520 nm as an indirect measurement of lipid accumulation. The inhibition rate (IR) of lipid accumulation (%) was calculated, as follows:

$$IR (\%) = (1- OD_{sample}/OD_{control}) \times 100\%$$

Determination of liproprotein lipase (LPL) activity *in vitro.* The cellular LPL activity was measured using a commercial lipoprotein lipase activity assay kit (Cell Biolabs, Inc, CA**,** USA). The assay utilizes a fluorogenic triglyceride analog as a lipase substrate, which in an uncleaved condition remains in a non-fluorescent, quenched state. However, when the substrate is hydrolyzed by LPL at the sn-1 position, a fluorescent product is formed which can be measured in a SpectraMax M3 fluorescence reader (excitation: 480-485 nm/emission: 515-525 nm with 495 nm cutoff). Briefly, 3T3-L1 and L6 cells were seeded at a density of 2 × 10^5 cells per well into 6-well plates and cultured overnight. The cells were then treated with URAM or FRAM at 100 and 200 μg/ml concentrations for 24 h. Following the treatment schedule, the media were collected and the cells were subjected to lysis. The LPL activity of both the lysates and media was determined according to the instructions provided by the kit manufacturer.

Determination of glucose uptake by L6 myocytes and insulin-resistant HepG2 cells *in vitro*. L6 myocytes and HepG2 cells were seeded at a density of 1 × 10^4 cells per well into 96-well black, clear bottom culture plates (Greiner Bio–One, Frickenhausen, Germany) and cultured overnight in glucose-containing DMEM supplemented with 10% FBS. In the case of L6 myocytes, the media were then discarded and after washing the cells with sterile PBS, pH 7.4, glucose-free DMEM supplemented with 10% FBS were added to the wells and the cells were incubated under this condition for 12 h. For HepG2 cells, after removal of the media, the cells were washed with PBS, pH 7.4, and then treated with 18 mM glucosamine (Sigma-Aldrich, MO, USA) in glucose-containing DMEM supplemented with 10% FBS for 24 h to induce insulin resistance. The media were then discarded and the cells were washed, followed by addition of glucose-free DMEM supplemented with 10% FBS to the wells and the cells were further incubated for 12 h. After completion of this glucose deprivation process of both cell types, the cells were treated with glucose-free DMEM, or 100 nM insulin (in glucose-free DMEM) alone or in combination with URAM or FRAM (in glucose-free DMEM) at 100 and 200 μg/ml concentrations for 12 h. The cells were then treated for 6 h with 2-deoxy-2-[(7-nitro-2,1,3-benzoxadiazol-4-yl)amino]-D-glucose (2-NBDG, Life Technologies, CA, USA), a fluorescent glucose analogue frequently used as a non-toxic probe for measurement of cellular glucose uptake activity, at a final concentration of 100 μM. Finally, the uptake of 2-NBDG by the cells was detected by fluorescence microscopy (Olympus BX-61, Tokyo, Japan) and determined using a SpectraMax M3 fluorescence reader (Molecular Devices, CA, USA) with excitation and emission wavelengths at 475 and 515 nm, respectively.

Determination of gene expression in 3T3-L1 cells using real-time PCR. Differentiated 3T3-L1 cells were treated with URAM or FRAMs at 100 and 200 μg/ml concentrations for 24 h. Following this, total RNA was isolated from the cells using TRIsure reagent (Bioline, MA, USA) and subsequently cDNA was reverse transcribed using an AccuPower RT premix kit (Bioneer, Daejeon, Korea) according to the product manual of the kit manufacturer. The real-time PCR amplification reaction was performed in a LightCycler instrument (Roche Applied Science, Indianapolis) using the listed primers (Table S3) and LightCycler® FastStart DNA Master SYBR Green kit (Roche Applied Science). The reaction was carried out following the instructions of the kit manufacturer in a total reaction volume of

20 µl containing PCR mix, 1 µl of cDNA, and gene-specific primers (10 pmol for each). The relative gene expression was represented by $2^{-\Delta Ct}$ using β-actin as a housekeeping gene for normalization, where C_t is the crossing threshold value and $\Delta C_t = C_t$ (target gene$_)$ - C_t (β-actin).

Determination of nitric oxide production *in vitro*. RAW 264.7 cells were grown at a density of 2 × 10^5 cells/well in 24-well plates. After incubation for 12 h, the cells were treated with FRAM or URAM (0, 50, 100, or 200 µg/ml) for 6 h and then exposed to lipopolysaccharide (LPS, from *Pseudomonas aeruginosa*, Sigma-Aldrich**)** final concentration of 0.2 µg/ml, pH 7.4) or DMEM for 24 h. The nitric oxide content in the supernatant was determined using the Griess reagent system kit (Promega, WI, USA) in accordance with the instructions of the kit manufacturer.

Determination of transepithelial electrical residence (TEER) *in vitro*. HTC 116 cells were seeded at a density of 2 × 10^5 cells per well onto the apical wells of Millicell-24 cell culture insert plates (12 mm in diameter 0.4 µm membrane pore size; Millipore, Bedford, MA, USA) and grown as monolayers. The cells were then treated with various concentrations (0, 100, 200, or 400 µg/ml) of URAM or FRAM for 24 h and exposed to either LPS (10 µg/ml) or culture medium for 12 h. Eventually, TEER was measured on the inside and outside of the apical wells using a Millicell ERS-2 epithelial volt-ohm meter (Millipore, MA, USA) according to the instructions of the manufacturer. The results are expressed as a percentage of the normal cells that did not receive herbal or LPS treatments.

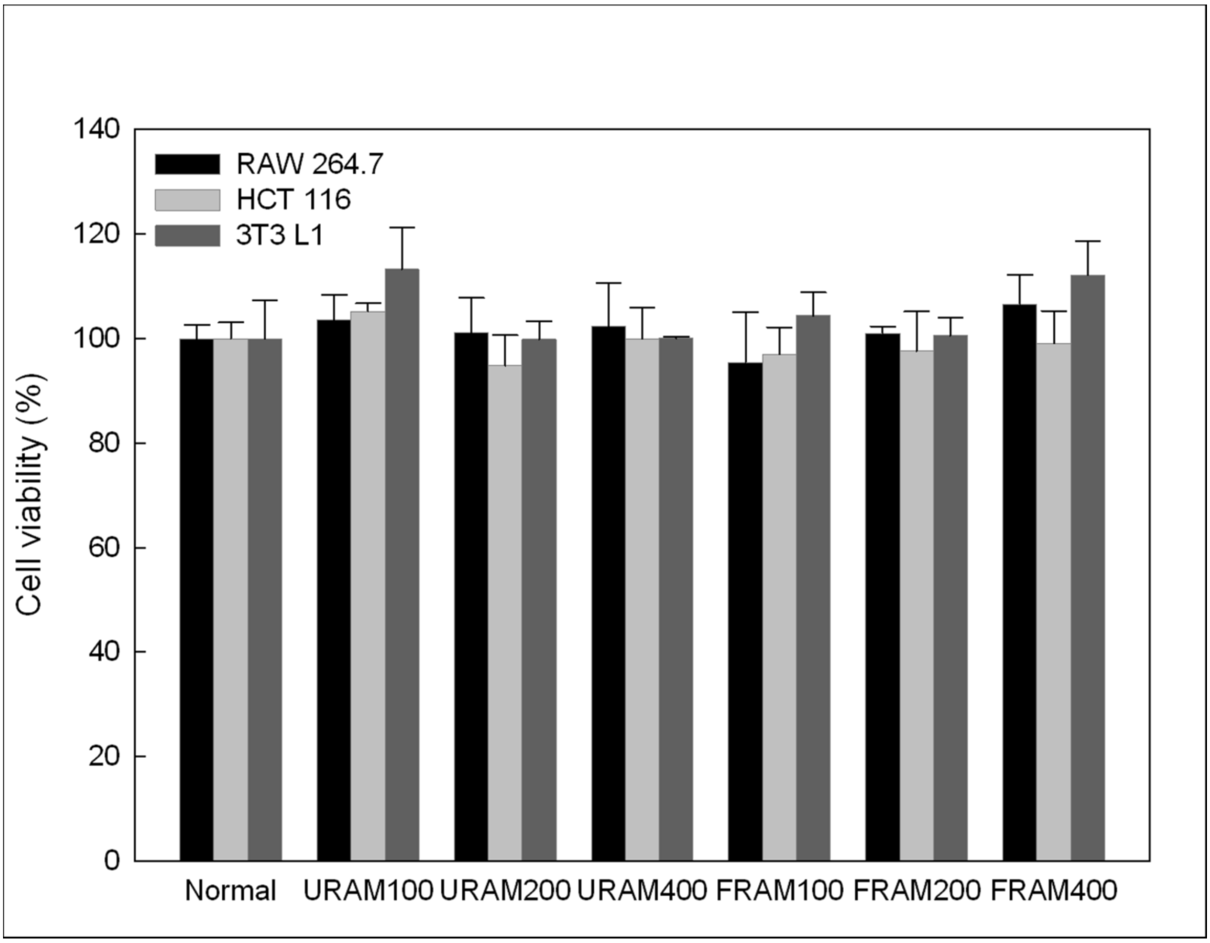

Fig. S1. The impact of URAM and FRAM on the viability of cells. RAW 264.7, HCT 116, and 3T3-L1 cells were treated with URAM or FRAM at the indicated concentrations for 24 h. The cells in the normal group were not treated. The detailed treatment conditions and experimental procedures are described in the Supplementary methods section. The viability of the cells in the normal group was set to 100%.The data are expressed as the mean ± SD, n = 3. No statistically significant differences in cell viability were observed compared to the normal.

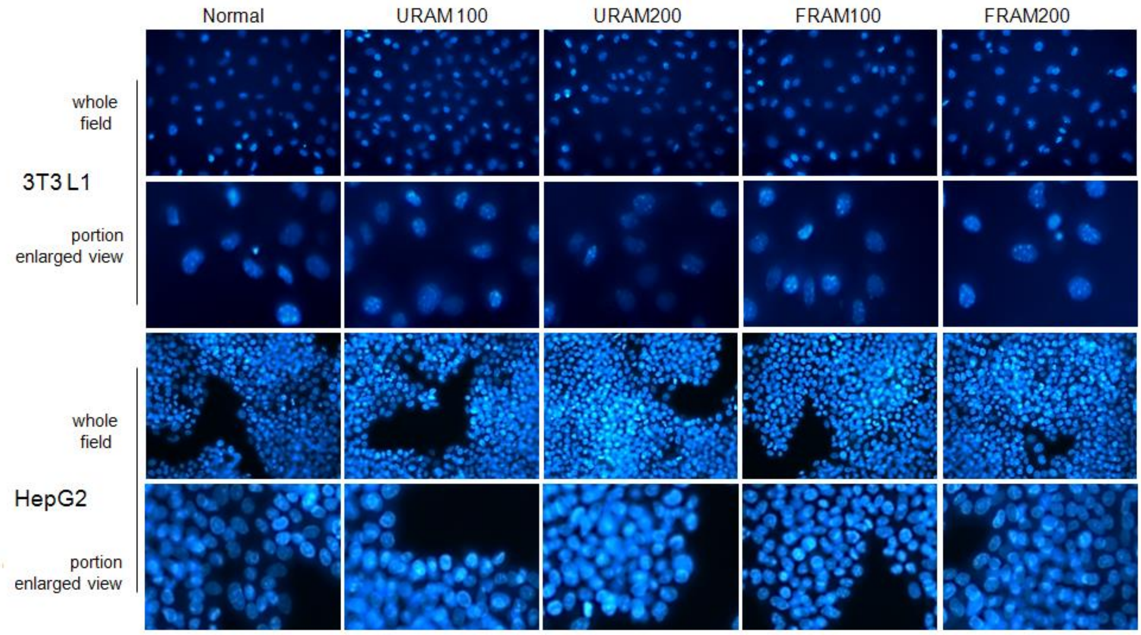

Fig. S2. URAM and FRAM did not induce apoptosis in 3T3-L1 or HepG2 cells. Cultured 3T3-L1 and HepG2 cells were treated with URAM or FRAM (100 or 200 μg/ml) for 24 h. The cells in the normal group were not treated. Following the treatment schedule, the cells were washed with PBS and then stained with PI (5 μg/ml, 10 min, 37°C) and Hoechst 33342 (15 μg/ml, 10 min, 4°C) successively. Finally, the cells were observed by fluorescence microscopy and images were captured. The detailed treatment conditions and experimental procedures are described in the Supplementary methods section.

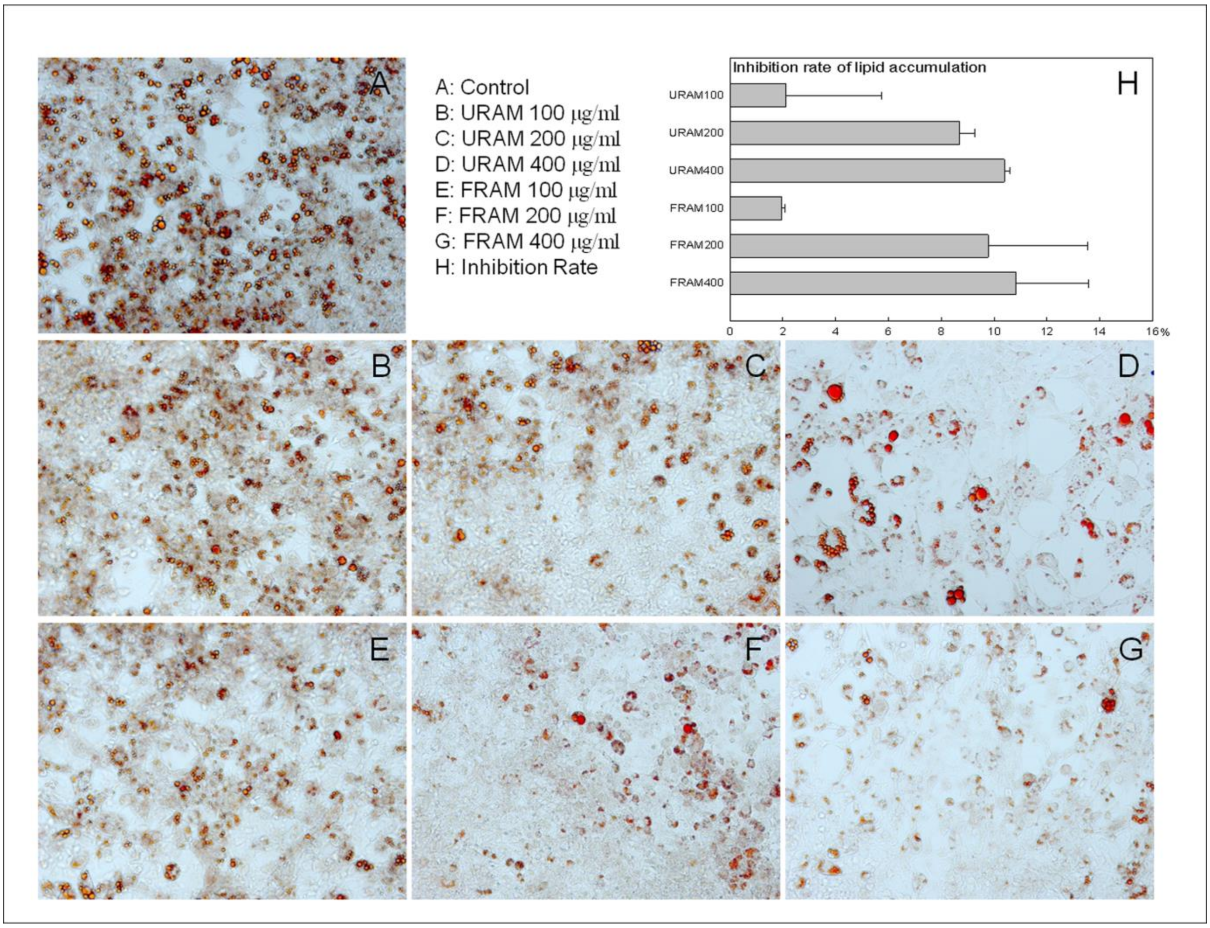

Fig. S3. The impact of URAM and FRAM on intracellular lipid accumulation in differentiated 3T3-L1 cells. After the termination of treatment with DMEM (control, A), URAM (B-D) or FRAM (E-G) for 24 h, the differentiated 3T3-L1 cells were fixed in 10% formalin and then washed with 60% isopropanol. Subsequently, the cells were stained with Oil red O and observed under an inverted light microscope for capture of images. The cells were then treated with 100% isopropanol and the absorbance of the resultant supernatant was read at 520 nm as an indirect measurement of lipid accumulation. Accordingly, the degree of inhibition of lipid accumulation in the different treatment groups was quantitated and expressed as the mean ± SD, n = 3 (H). The detailed treatment conditions and experimental procedures are described in the Supplementary methods section.

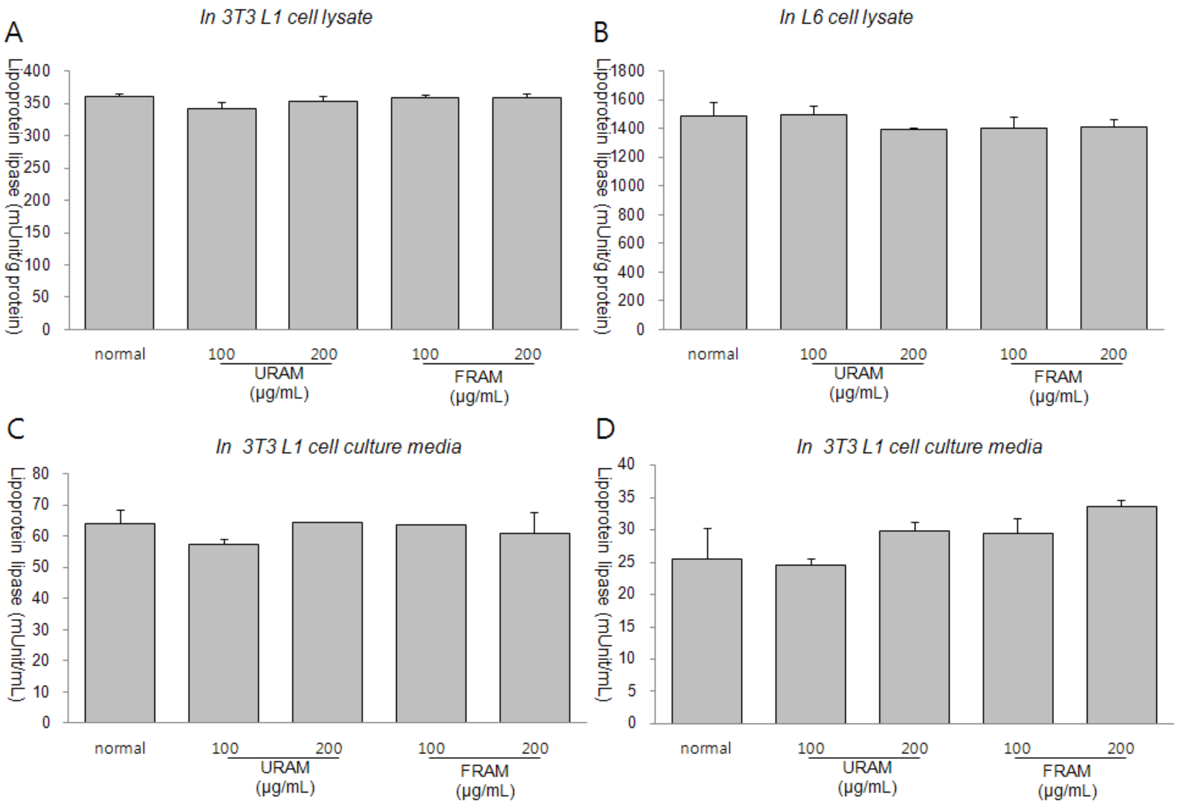

Fig. S4. Imapct of URAM and FRAM on the enzyme activity of lipoprotein lipase in 3T3-L1 or L6 cells. Cultured 3T3-L1 and L6 cells were treated with URAM or FRAM (100 or 200 μg/ml) for 24 h. The cells in the normal group were not treated. Following the treatment schedule, the media were collected and the cells were lysed. LPL activity in both cell lysates and culture media was quantitatively determined. The detailed treatment conditions and experimental procedures are described in the Supplementary methods section. The data are expressed as the mean ± SD, (n=3). No statistically significant differences were observed in the treated groups compared to the normal.

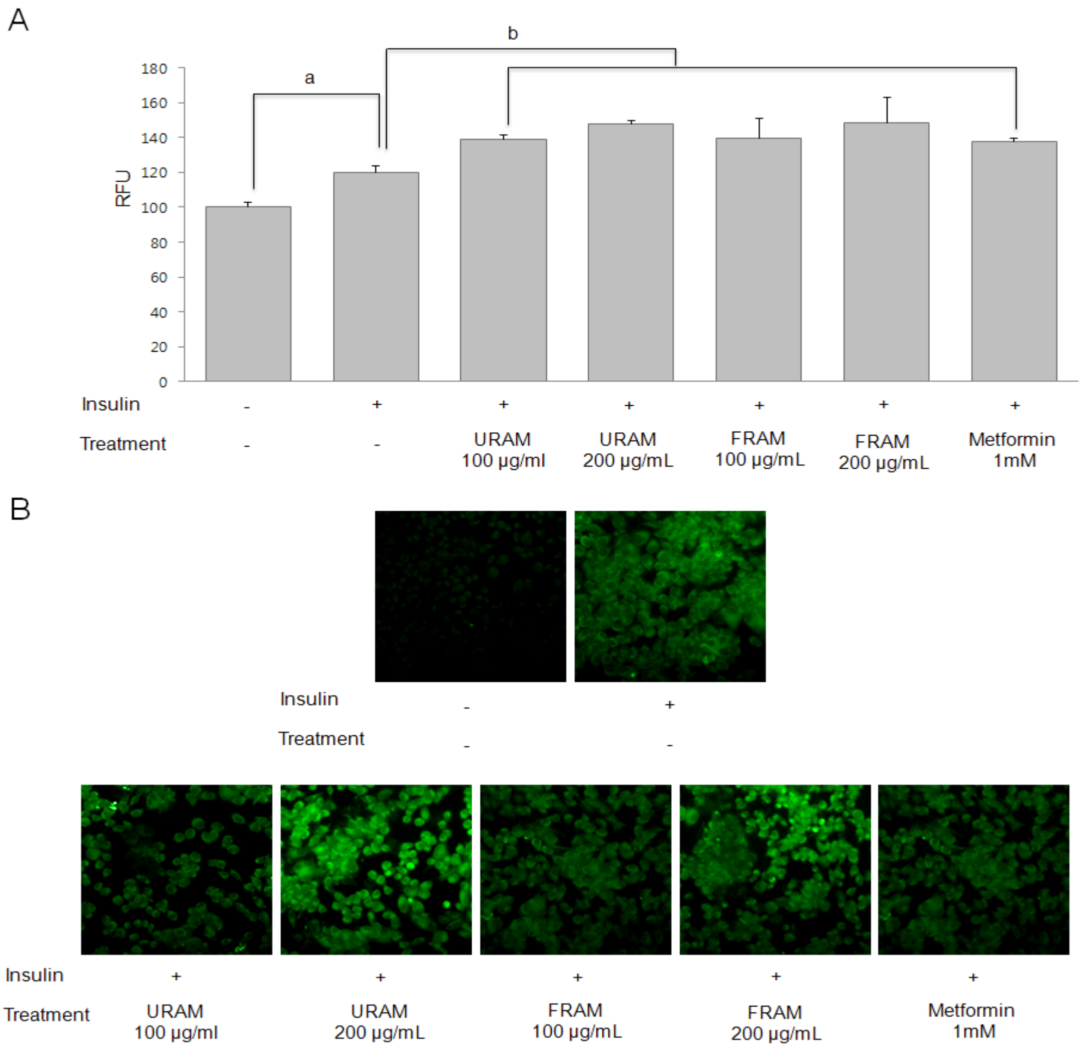

Fig. S5. The impact of URAM and FRAM on the glucose uptake by L6 cells. After washing with PBS, cultured L6 cells were subjected to glucose deprivation by incubation in glucose-free media for 12 h. The cells were then treated with 100 nM of insulin in absence or presence of URAM or FRAM (100 or 200 μg/ml) in glucose-free media for 12 h. Metformin (1mM) was used as a positive control. Following the treatment schedule, 2-NBDG was added to the media at a final concentration of 100 μM and the cells were incubated in this condition for 6 h. Finally, the fluorescence intensity was determined via both fluorescence microscope and

spectrofluorophotometer. The detailed treatment conditions and experimental procedures are described in the Supplementary methods section. Data are expressed as the mean ± SD (n=3). Superscript letters (a or b) are significantly different ($P < 0.05$) according to post hoc one way ANOVA analysis. NS means non-significant.

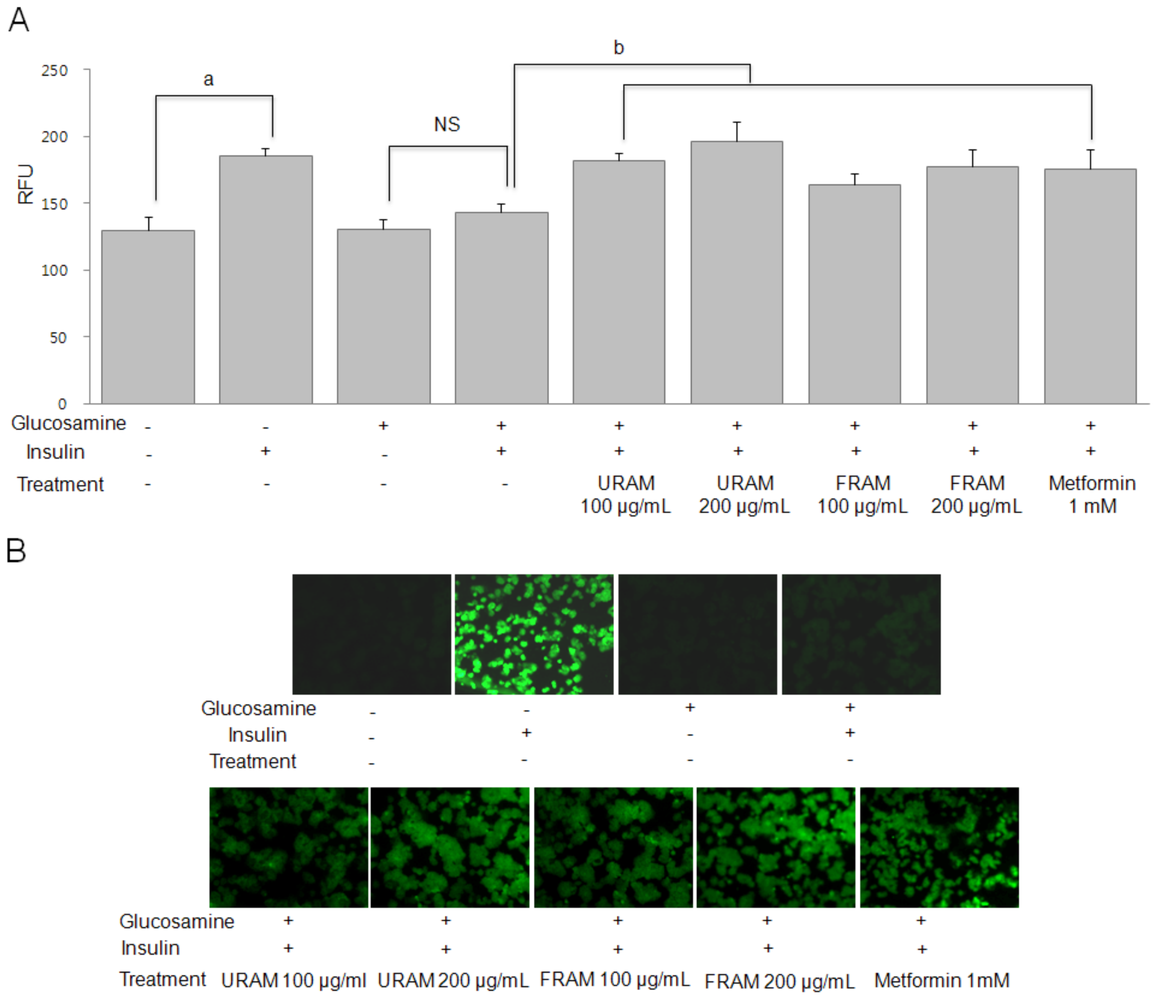

Fig. S6. The impact of URAM and FRAM on glucosamine-induced insulin resistance in HepG2 cells. Cultured HepG2 cells were treated with glucosamine (18 mM) for 24 h to induce insulin resistance. After washing with PBS, the cells were subjected to glucose deprivation by incubation in glucose-free media for 12 h. The cells were then treated with 100 nM of insulin in absence or presence of URAM or FRAM (100 or 200 μg/ml) in glucose-free media for 12 h. Metformin (1mM) was used as a positive control. Following the treatment schedule, 2-NBDG was added to the media at a final concentration of 100 μM and the cells were incubated in this

condition for 6 h. Finally, the fluorescence intensity was determined via both fluorescence microscope and spectrofluorophotometer. The detailed treatment conditions and experimental procedures are described in the Supplementary methods section. Data are expressed as the mean ± SD (n=3). Superscript letters (a or b) are significantly different ($P < 0.05$) according to post hoc one way ANOVA analysis. NS means non-significant.

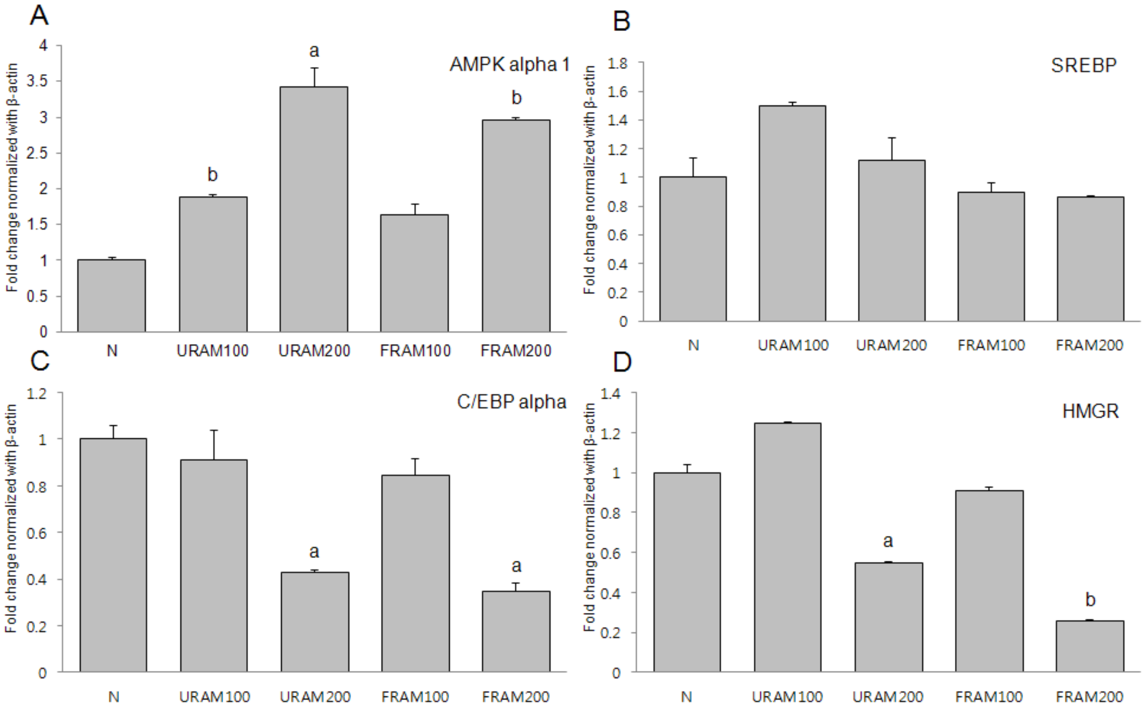

Fig. S7. The impact of URAM and FRAM on the gene expression of vital transcriptional factors in differentiated 3T3-L1 cells. Differentiated 3T3-L1 cells were treated with URAM and FRAM (100 or 200 μg/ml) for 6 h, followed by total RNA isolation, cDNA reverse transcription, and real-time PCR amplification, in order. The detailed treatment conditions and experimental procedures are described in the Supplementary methods section. Data are expressed as the mean ± SD (n=4). a, b, significantly different compared to normal ($^{a}P < 0.05$ or $^{b}P < 0.01$) according to post hoc one way ANOVA analysis.

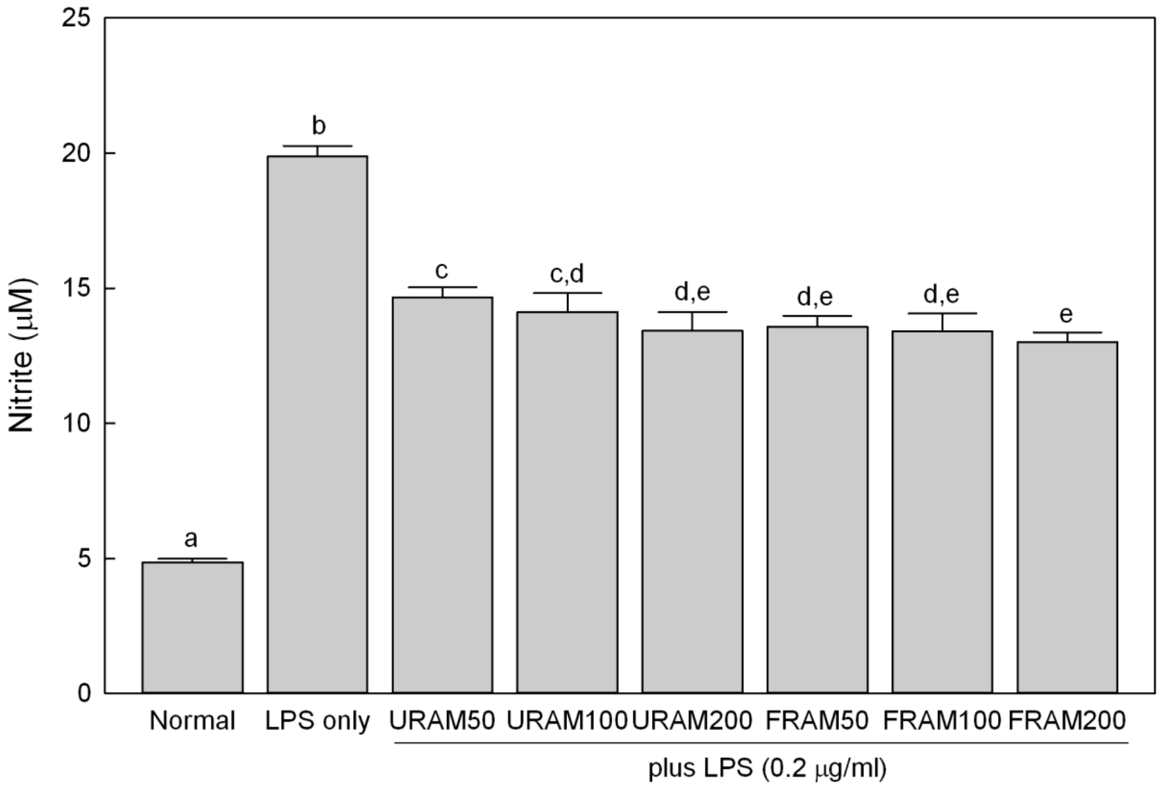

Fig. S8. Inhibition of LPS-induced NO production by URAM and FRAM in RAW 264.7 cells. Immediately after pretreatment with URAM or FRAM at 0 (for normal and LPS-alone groups, received DMEM instead of herbal extract), 50, 100, and 200 μg/ml doses for 6 h, RAW 264.7 cells were exposed to DMEM (normal) or 0.2 μg/ml LPS for 24 h followed by measurement of NO as described in the Supplementary methods section. Data are expressed as the mean ± SD (n=3). Data with different letters are significantly different ($P < 0.05$) according to post hoc one way ANOVA analysis.

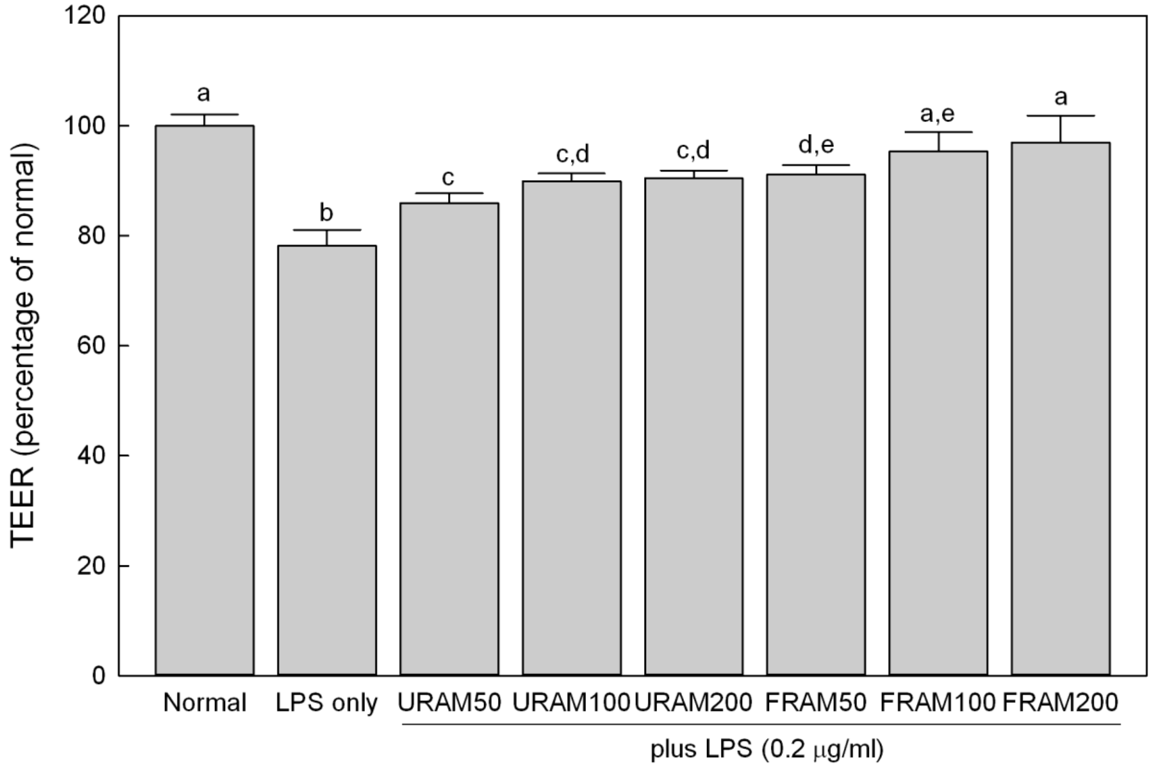

Fig. S9. The impact of URAM and FRAM on the membrane permeability of LPS-treated HCT 116 cells. Immediately after pretreatment with URAM or FRAM at 0 (normal and LPS-alone groups, received McCoy's 5A medium instead of herbal extract), 50, 100, and 200 μg/ml doses for 24 h, HCT 116 cells were exposed to McCoy's 5A medium (normal) or 10 μg/ml LPS for 24 h followed by measurement of transepithelial electrical residence (TEER) as described in the Supplementary methods section. Data are expressed as the mean ± SD (n=3). Data with different letters are significantly different (P < 0.05) according to post hoc one way ANOVA analysis.

Table S1. Diets formulation and ingredients

Formulation	Normal diet (AIN-93G)		High Fat Diet	
	gm%	kcal%	gm%	kcal%
Protein	20	20	27	20
Carbohydrate	64	64	25	20
Fat	7	16	36	60
Kcal/kg	4,000		5,333	

Ingredient	g	kcal	g	kcal
Casein (from milk)	200	800	200	800
Corn starch	397,486	1,590	47,536	190
Sucrose	100	400	0	0
Dextrose	132	528	132	528
Cellulose	50	0	50	0
Soybean oil	70	630	25	225
Lard	0	0	245	2,205
Mineral mixture	35	0	35	0
Vitamin mixture	10	40	10	40
TBHQ	0.014	0	0.014	0
L-Cystine	3	12	3	12
Choline bitartrate	2.5	0	2.5	0
Total	1,000	4,000	750.1	4,000

Abbreviations: TBHQ, tertiary butylhydroquinone

Table S2. Primer sequences used for gut microbiota analysis by real-time PCR

Bacteria	Primer sequence	OAT	Ref.
Lactobacillus spp.	5'-GAG GCA GCA GTA GGG AAT CTT C-3'<br>5'-GGC CAG TTA CTA CCT CTA TCC TTC TTC-3'	60°C	[S1]
Bifdobacterium spp.	5'-CGC GTC TGG TGT CAA AG-3'<br>5'-CCC CAC ATC CAG CAT CCA-3'	65°C	[S2]
Akkermansia spp.	5'-CAG CAC GTG AAG GTG GGG AC-3'<br>5'-CCT TGC GGT TGG CTT CAG AT-3'	60°C	[S3]
Bacteroidetes	5'-GGA RCA TGT GGT TTA ATT CGA TGA T-3'<br>5'-AGC TGA CGA CAA CCA TGC AG-3'	66°C	[S4]
Firmicutes	5'-GGA GYA TGT GGT TTA ATT CGA AGC A-3'<br>5'-AGC TGA CGA CAA CCA TGC AC-3'	69°C	[S4]

Abbreviations: OAT, optimized annealing temperature; Ref., references

Table S3. Primer sequences used for gene expression analysis *in vitro* by real-time PCR

Gene name	Primer sequence	OAT	Ref.
AMPK α1	5'- AAG CCG ACC CAA TGA CAT CA -3'<br>5'- CTT CCT TCG TAC ACG CAA AT -3'	53°C	[S5]
SREBP	5'- GCT GGT TTG ACT GGA TGG TT -3'<br>5'- ACC TTT GGC GAG GTC TAG GT -3'	58°C	AF374266.1*
C/EBP α	5'- TCG GTG GAC AAG AAC AGC AA -3'<br>5'- CGG TCA TTG TCA CTG GTC AAC -3'	57°C	BC058161.1*
HMGCR	5'- GTA AGC GCA GTT CCT TCC GC -3'<br>5'- TTG TAG CCT CAC AGT CCT TGG -3'	58°C	NM_008255.2#
β-actin	5'- GCA AGT GCT TCT AGG CGG AC -3'<br>5'- AAG AAA GGG TGT AAA ACG CAG C -3'	53°C	NM_007393.3#

Abbreviations: OAT, optimized annealing temperature; Ref., references; AMPK α1, 5' adenosine monophosphate-activated protein kinase alpha 1; SREBP, sterol regulatory element binding protein; C/EBP α, CCAAT-enhancer-binding protein alpha; HMGCR, 3-hydroxy-3-methyl-glutaryl-CoA reductase; β-actin, beta-actin.

Notes: superscript symbol (* or #) means target gene accession No. from GenBank or NCBI.

Supplementary references

S1. Cani PD, *et al.* Changes in gut microbiota control metabolic endotoxemia-induced inflammation in high-fat diet-induced obesity and diabetes in mice. *Diabetes* **57**, 1470-1481 (2008).

S2. Delroisse JM, Boulvin AL, Parmentier I, Dauphin RD, Vandenbol M, Portetelle D. Quantification of Bifidobacterium spp. and Lactobacillus spp. in rat fecal samples by real-time PCR. *Microbiol Res* **163**, 663-670 (2008).

S3. Everard A, *et al.* Cross-talk between Akkermansia muciniphila and intestinal epithelium controls diet-induced obesity. *Proc Natl Acad Sci U S A* **110**, 9066-9071 (2013).

S4. Guo X, Xia X, Tang R, Zhou J, Zhao H, Wang K. Development of a real-time PCR method for Firmicutes and Bacteroidetes in faeces and its application to quantify intestinal population of obese and lean pigs. *Lett Appl Microbiol* **47**, 367-373 (2008).

S5. Mulligan JD, Gonzalez AA, Stewart AM, Carey HV, Saupe KW. Upregulation of AMPK during cold exposure occurs via distinct mechanisms in brown and white adipose tissue of the mouse. *J Physiol* **580**, 677-684 (2007).
